# Supplementary material for: The DCM Project Portal: A direct-to-participant platform of The DCM Research Project
Source: medRxiv. 2023 Jun 29:2023.06.22.23291764. Preprint. [Version 1] doi: 10.1101/2023.06.22.23291764 (PMC10327249; doi:10.1101/2023.06.22.23291764)
Supplement: Supplement 1 [file NIHPP2023.06.22.23291764v1-supplement-1.pdf]

medRxiv preprint doi: https://doi.org/10.1101/2023.06.22.23291764; this version posted June 29, 2023. The copyright holder for this preprint (which was not certified by peer review) is the author/funder, who has granted medRxiv a license to display the preprint in perpetuity.
It is made available under a CC-BY 4.0 International license .

Supplemental Material

Inclusion/Exclusion Criteria for probands in the DCM Precision Medicine Study

All probands have met diagnostic criteria for idiopathic DCM: left ventricular ejection fraction <50%; left ventricular enlargement (echo-derived left ventricular end-diastolic dimension ≥95th percentile for gender/height). In addition, they were in one of the target ethnicity-ancestry groups, were able to communicate in English (or in Spanish at sites approved to recruit individuals of Hispanic ethnicity), were able to give informed consent (or assent and parental consent for children), and were willing and able to participate in a family-based study. Relatives also were expected to satisfy these same requirements except for meeting diagnostic criteria for idiopathic DCM. Risk factors considered conventional for DCM, such as obesity, routinely treated hypertension, alcohol use/abuse, peripartum cardiomyopathy, or the presence of left-ventricular non-compaction, were not used as exclusion criteria.

Exclusion criteria included: Coronary artery disease (CAD) causing ischemic cardiomyopathy (>50% narrowing, any major epicardial coronary artery; clinical testing is indicated to exclude CAD routinely in (1) males >40 years or females >45 years without risk factors and (2) males or females >30 years with risk factors); Primary valvular disease; Cardiotoxic drug exposure, including Adriamycin and other cancer chemotherapeutics; Other forms of cardiomyopathy (e.g., hypertrophic cardiomyopathy, arrhythmogenic right ventricular cardiomyopathy, restrictive cardiomyopathy, Chagas cardiomyopathy); Congenital/structural heart disease; Sarcoid; Amyloid; Iron overload; Other active multisystem disease that may plausibly cause DCM (e.g., hypereosinophilic syndrome, cardiac involvement with connective tissue disease, Loeffler's endocarditis, endomyocardial fibrosis); Severe and untreated or untreatable hypertension (defined as systolic blood pressures routinely >180 mm Hg and/or

medRxiv preprint doi: https://doi.org/10.1101/2023.06.22.23291764; this version posted June 29, 2023. The copyright holder for this preprint (which was not certified by peer review) is the author/funder, who has granted medRxiv a license to display the preprint in perpetuity.
It is made available under a CC-BY 4.0 International license .

diastolic blood pressures >120 mm Hg. Untreated hypertension is an individual receiving no medications, and untreatable hypertension is an individual with severe hypertension persisting despite multidrug regimens and/or associated with other multisystem disease (e.g., scleroderma, other vasculitides, etc)); Exclusion beyond a reasonable doubt of all other detectable causes of cardiomyopathy (other than genetic) at the time of primary DCM diagnosis using a rigorous clinical standard congruent with an expert approach rendered by a board-certified heart failure/transplant cardiologist.

medRxiv preprint doi: https://doi.org/10.1101/2023.06.22.23291764; this version posted June 29, 2023. The copyright holder for this preprint (which was not certified by peer review) is the author/funder, who has granted medRxiv a license to display the preprint in perpetuity.
It is made available under a CC-BY 4.0 International license .

Tables S1-S5. Reported access to the internet in the United States population by generational age group and self-identified race and Hispanic ethnicity

To estimate internet connectivity in the population by self-identified race, ethnicity, generational age groups, data was exported from the Public Use Microdata Sample (PUMS) using the US Census Bureau's Microdata Access Tool (MDAT). ACS 1-Year Estimates PUMS was selected with the corresponding year to generate the dataset. Tables were generated by selecting the "Access to Internet" variable and the aforementioned demographic variables. "Age" was customized to captured to encompass age groups defined by birth years within generations (Generation Z: 1997-2012, Millennials: 1981-86, Generation X: 1965-80, Baby Boomers: 1946-64, Silent Generation: 1928-45, WWII generation: 1901-1927). Data were exported for each variable combination for both 2019 and 2021 to determine if there was a reported increase between the two years as a possible impact of the COVID-19 pandemic on internet connectivity.

Table S1. Total ACS 1-Year Estimates Access to the Internet in 2019 and 2021 According to the Public Use Microdata Sample

**Access to the Internet in the United States (2019)**		
	2019	2021
N/A[a]	8084362 (2.5%)	7760859 (2.3%)
Yes, Total (with or without paying a service[b]*)*	295581562 (90.0%)	308066247 (92.8%)
Yes, by paying a service	288656692 (87.9%)	301737502 (90.9%)
Yes, without paying a service	6924870 (2.1%)	6328745 (1.9%)
No access[c]	24573599 (7.5%)	16066639 (4.8%)
**Total**	328239523 (100%)	331893745 (100%)

[a]N/A refers to individuals who lived in group quarters or housing units that were vacant at the time of data collection
[b]service refers to paying a cell phone company of internet service provider
[c]No responses are regarding to access at home; may not be representative of access to public internet sources

Table S2. Reported Internet Access in the US Population in 2019 and 2021 According to Census Data

	2019 (%)					2021 (%)					
	Yes, Total (with or without paying a service)	Yes, by paying a service[b]	Yes, without paying a service	No Access[c]	N/A[d]	Yes, Total (with or without paying a service)	Yes, by paying a service[b]	Yes, without paying a service	No Access[c]	N/A[d]	Change in Reported Internet Access between 2019 and 2021
**Generational Age Groups[a]** (generation name, corresponding years of birth listed)											
Generation Z (2012-1997)	90.69%	88.53%	2.16%	4.54%	4.77%	92.68%	90.74%	1.94%	2.41%	4.91%	+1.99%
Millennials (1996-1981)	93.03%	91.02%	2.01%	4.67%	2.30%	95.28%	93.62%	1.65%	2.68%	2.04%	+2.24%
Generation X (1980-1965)	92.88%	91.03%	1.85%	5.49%	1.63%	94.88%	93.16%	1.72%	3.60%	1.52%	+2.00%
Baby Boomer (1964-1946)	88.58%	86.46%	2.12%	9.96%	1.46%	91.06%	88.98%	2.08%	7.56%	1.38%	+2.48%
Silent Generation (1945-1928)	73.72%	70.72%	2.99%	22.75%	3.53%	77.91%	74.79%	3.12%	18.16%	3.92%	+4.20%
**Self-identified Race**											
Black	84.35%	81.44%	2.91%	11.36%	4.29%	89.03%	86.54%	2.50%	6.64%	4.32%	+4.68%
White	90.80%	88.77%	2.03%	6.99%	2.21%	93.09%	91.30%	1.78%	4.66%	2.25%	+2.29%
**Self-identified Ethnicity**											
Non-Hispanic	90.28%	88.22%	2.06%	7.12%	2.60%	92.71%	90.86%	1.86%	4.78%	2.51%	+2.43%
Hispanic	89.02%	86.69%	2.33%	9.12%	1.87%	93.28%	91.16%	2.12%	5.11%	1.61%	+4.26%

[a]only showing generations including those aged ≥18 years as of 2023 sorted by generational name and years of birth.
[b]service refers to paying a cell phone company of internet service provider
[c]No responses are regarding to access at home; may not be representative of access to public internet sources
[d]N/A refers to individuals who lived in group quarters or housing units that were vacant at the time of data collection

Table S3a. ACS 1-Year Estimates Access to the Internet in 2019 According to the Public Use Microdata Sample by Generational Age Group

**2019 Internet Usage by Generational Cohorts**					
**Source: ACS 1-Year Estimates Public Use Microdata Sample 2019**					
Weight used: PWGTP					
**Access to the Internet (ACCESS)**					
**Generational Age Groups[a]** (generation name, corresponding years of birth listed)	Total	N/A[b]	Yes, by paying a service[c]	Yes, without paying a service	No access[d]
**Total[a]**	328239523	8084362 (2.46%)	288656692 (87.94%)	6924870 (2.11%)	24573599 (7.49%)
**Generation Z (2012-1997)**	67805245	3231114 (4.77%)	60026497 (88.53%)	1465901 (2.16%)	3081733 (4.54%)
**Millennials (1996-1981)**	71525288	1641951 (2.30%)	65104337 (91.02%)	1438565 (2.01%)	3340435 (4.67%)
**Generation X (1980-1965)**	65308606	1063134 (1.63%)	59447159 (91.03%)	1210772 (1.85%)	3587541 (5.49%)
**Baby Boomer (1964-1946)**	71803346	1047398 (1.46%)	62078234 (86.46%)	1523892 (2.12%)	7153822 (9.96%)
**Silent Generation (1945-1928)**	22850856	806891 (3.53%)	16160835 (70.72%)	683925 (2.99%)	5199205 (22.75%)
**WWII (1901-1924)[e]**	1880765	278439 (14.8%)	862494 (45.86%)	85710 (4.56%)	654122 (34.78%)

[a]only showing generations including those aged ≥18 years as of 2023 sorted by generational name and years of birth. Total also includes the younger Generation Alpha which does not include individuals aged ≥18 years as of 2023.
[b]N/A refers to individuals who lived in group quarters or housing units that were vacant at the time of data collection
[c]service refers to paying a cell phone company of internet service provider
[d]No responses are regarding to access at home; may not be representative of access to public internet sources
[e]99 is the upper age limit of the Census data and not reflective of the actual age cutoff of this generation

Table S3b. ACS 1-Year Estimates Access to the Internet in 2021 According to the Public Use Microdata Sample by Generational Age Group

2021 Internet Usage by Generational Cohorts					
Source: ACS 1-Year Estimates Public Use Microdata Sample 2021					
Weight used: PWGTP					
Access to the Internet (ACCESSINET)					
**Generational Age Groups[a]** (generation name, corresponding years of birth listed)	Total	N/A[b]	Yes, by paying a service[c]	Yes, without paying a service	No access[d]
**Total[a]**	331893745	7760859 (2.34%)	301737502 (90.91%)	6328745 (1.91%)	16066639 (4.84%)
**Generation Z (2012-1997)**	69075253	3394180 (4.91%)	62680388 (90.74%)	1339025 (1.94%)	1661660 (2.41%)
**Millennials (1996-1981)**	72109485	1471669 (2.04%)	67512342 (93.62%)	1191720 (1.65%)	1933754 (2.68%)
**Generation X (1980-1965)**	65748113	1000636 (1.52%)	61252802 (93.16%)	1127977 (1.72%)	2366698 (3.60%)
**Baby Boomer (1964-1946)**	70571030	975402 (1.38%)	62796417 (88.98%)	1466078 (2.08%)	5333133 (7.56%)
**Silent Generation (1945-1928)**	18692329	733138 (3.92%)	13980278 (74.79%)	583471 (3.12%)	3395442 (18.16%)
**WWII (1901-1924)[e]**	1168943	164797 (14.1%)	611421 (52.31%)	57470 (4.92%)	335255 (28.68%)

[a]only showing generations including those aged $\geq$18 years as of 2023 sorted by generational name and years of birth. Total also includes the younger Generation Alpha which does not include individuals aged $\geq$18 years as of 2023.
[b]N/A refers to individuals who lived in group quarters or housing units that were vacant at the time of data collection
[c]service refers to paying a cell phone company of internet service provider
[d]No responses are regarding to access at home; may not be representative of access to public internet sources
[e]99 is the upper age limit of the Census data and not reflective of the actual age cutoff of this generation

medRxiv preprint doi: https://doi.org/10.1101/2023.06.22.23291764; this version posted June 29, 2023. The copyright holder for this preprint (which was not certified by peer review) is the author/funder, who has granted medRxiv a license to display the preprint in perpetuity.
It is made available under a CC-BY 4.0 International license .

Table S4a. ACS 1-Year Estimates Access to the Internet in 2019 According to the Public Use Microdata Sample by Self-identified Race

2019 Internet Usage by Race					
Source: ACS 1-Year Estimates Public Use Microdata Sample 2019					
Weight used: PWGTP					
Access to the Internet (ACCESS)					
**Recoded detailed race code**	Total	N/A[a]	Yes, by paying a service[b]	Yes, without paying a service	No access[c]
Total	328239523	8084362 (2.46%)	288656692 (87.94%)	6924870 (2.11%)	24573599 (7.49%)
White	236532194	5233723 (2.21%)	209980189 (88.77%)	4794213 (2.03%)	16524069 (6.99%)
Black or African American	42043031	1802226 (4.29%)	34241467 (81.44%)	1223914 (2.91%)	4775424 (11.36%)
American Indian	2248236	66416 (2.95%)	1718308 (76.43%)	64888 (2.89%)	398624 (17.73%)
Alaska Native	112956	3611 (3.20%)	90278 (79.92%)	1199 (1.06%)	17868 (15.82%)
American Indian and Alaska Native tribes specified; or American Indian or Alaska Native, not specified and no other races	480428	29074 (6.05%)	363711 (75.71%)	11995 (2.50%)	75648 (15.75%)
Asian	18608918	369549 (1.99%)	17384042 (93.42%)	243401 (1.31%)	611926 (3.29%)
Native Hawaiian and Other Pacific Islander	610822	21488 (3.52%)	520773 (85.26%)	12008 (1.97%)	56553 (9.26%)
Some other race	16266146	284217 (1.75%)	14005776 (86.1%)	367539 (2.26%)	1608614 (9.89%)
Two or More Races	11336792	274058 (2.42%)	10352148 (91.31%)	205713 (1.81%)	504873 (4.45%)

[a]N/A refers to individuals who lived in group quarters or housing units that were vacant at the time of data collection
[b]service refers to paying a cell phone company of internet service provider
[c]No responses are regarding to access at home; may not be representative of access to public internet sources

medRxiv preprint doi: https://doi.org/10.1101/2023.06.22.23291764; this version posted June 29, 2023. The copyright holder for this preprint (which was not certified by peer review) is the author/funder, who has granted medRxiv a license to display the preprint in perpetuity.
It is made available under a CC-BY 4.0 International license .

Table S4b. ACS 1-Year Estimates Access to the Internet in 2021 According to the Public Use Microdata Sample by Self-identified Race

2021 Internet Usage by Race					
Source: ACS 1-Year Estimates Public Use Microdata Sample 2021					
Weight used: PWGTP					
Access to the Internet (ACCESSINET)					
**Recoded detailed race code**	Total	N/A[a]	Yes, by paying a service[b]	Yes, without paying a service	No access[c]
Total	331893745	7760859 (2.34%)	301737502 (90.91%)	6328745 (1.91%)	16066639 (4.84%)
White	202954389	4570818 (2.25%)	185306842 (91.30%)	3620575 (1.78%)	9456154 (4.66%)
Black or African American	40217512	1739001 (4.32%)	34802991 (86.54%)	1003596 (3.50%)	2671924 (6.64%)
American Indian	2562058	44465 (1.74%)	2193725 (85.62%)	81593 (3.18%)	242275 (9.46%)
Alaska Native	124111	5162 (4.16%)	102668 (82.72%)	3110 (2.51%)	13171 (10.61%)
American Indian and Alaska Native tribes specified; or American Indian or Alaska Native, not specified and no other races	475644	37044 (7.79%)	384921 (80.93%)	13027 (2.74%)	40652 (8.55%)
Asian alone	19174507	330048 (1.72%)	18196497 (94.90%)	236779 (1.23%)	411183 (2.14%)
Native Hawaiian and Other Pacific Islander	623315	17319 (2.78%)	556979 (89.36%)	20497 (3.29%)	28520 (4.58%)
Some other race	23899490	330223 (1.38%)	21666481 (90.66%)	548625 (2.30%)	1354161 (5.67%)
Two or More Races	41862719	686779 (1.64%)	38526398 (92.03%)	800943 (1.91%)	1848599 (4.42%)

[a]N/A refers to individuals who lived in group quarters or housing units that were vacant at the time of data collection
[b]service refers to paying a cell phone company of internet service provider
[c]No responses are regarding to access at home; may not be representative of access to public internet sources

medRxiv preprint doi: https://doi.org/10.1101/2023.06.22.23291764; this version posted June 29, 2023. The copyright holder for this preprint (which was not certified by peer review) is the author/funder, who has granted medRxiv a license to display the preprint in perpetuity.
It is made available under a CC-BY 4.0 International license .

Table S5a. ACS 1-Year Estimates Access to the Internet in 2019 According to the Public Use Microdata Sample by Hispanic Ethnicity

Source: ACS 1-Year Estimates Public Use Microdata Sample 2019					
Weight used: PWGTP					
Access to the Internet (ACCESS)					
**Recoded detailed Hispanic origin**	Total	N/A[a]	Yes, by paying a service[b]	Yes, without paying a service	No access[c]
**-> Total**	328239523	8084362 (2.46%)	288656692 (87.94%)	6924870 (2.11%)	24573599 (7.49%)
**Not Spanish/Hispanic/Latino**	267754746	6956137 (2.60%)	236221124 (88.22%)	5517231 (2.06%)	19060254 (7.12%)
**Total Spanish/Hispanic/Latino[4]**	60484777	1128225 (1.87%)	52435568 (86.69%)	1407639 (2.33%)	5513345 (9.12%)
**Mexican**	37185876	598560 (1.61%)	32218744 (86.64%)	851890 (2.29%)	3516682 (9.46%)
**Puerto Rican**	5846497	143254 (2.45%)	5118425 (87.55%)	138258 (2.36%)	446560 (7.64%)
**Cuban**	2381954	41861 (1.76%)	2058265 (86.41%)	68368 (2.87%)	213460 (8.96%)
**Dominican**	2085488	32331 (1.55%)	1836537 (88.06%)	54849 (2.63%)	161771 (7.76%)
**Costa Rican**	169269	3649 (2.16%)	153764 (90.84%)	3707 (2.19%)	8149 (4.81%)
**Guatemalan**	1655606	17506 (1.06%)	1324030 (79.97%)	46549 (2.81%)	267521 (16.16%)
**Honduran**	1073904	18338 (1.71%)	886382 (82.54%)	25169 (2.34%)	144015 (13.41%)
**Nicaraguan**	432579	4976 (1.15%)	379299 (87.68%)	12001 (2.77%)	36303 (8.39%)
**Panamanian**	193274	4156 (2.15%)	178062 (92.13%)	1661 (0.86%)	9395 (4.86%)
**Salvadoran**	2343993	26518 (1.13%)	2041153 (87.08%)	45662 (1.95%)	230660 (9.84%)
**Other Central American**	59512	646 (1.09%)	49894 (83.84%)	962 (1.62%)	8010 (13.46%)
**Argentinean**	308116	5164 (1.68%)	291457 (94.59%)	5278 (1.71%)	6217 (2.02%)
**Bolivian**	135191	348 (0.26%)	127359 (94.21%)	1614 (1.19%)	5870 (4.34%)
**Chilean**	158927	2746 (1.73%)	152558 (95.99%)	1680 (1.06%)	1943 (1.22%)
**Colombian**	1238369	15478 (1.25%)	1157582 (93.48%)	22005 (1.78%)	43304 (3.50%)
**Ecuadorian**	707719	9297 (1.31%)	643842 (90.97%)	12676 (1.79%)	41904 (5.92%)
**Paraguayan**	30061	89 (0.30%)	29444 (97.95%)	351 (1.17%)	177 (0.59%)
**Peruvian**	652465	7505 (1.15%)	601660 (92.21%)	12717 (1.95%)	30583 (4.69%)
**Uruguayan**	73233	201 (0.27%)	70483 (96.24%)	608 (0.83%)	1941 (2.65%)
**Venezuelan**	538536	4993 (0.93%)	490070 (91.00%)	13037 (2.42%)	30436 (5.65%)
**Other South American**	24318	183 (0.75%)	21978 (90.38%)	521 (2.14%)	1636 (6.73%)

medRxiv preprint doi: https://doi.org/10.1101/2023.06.22.23291764; this version posted June 29, 2023. The copyright holder for this preprint (which was not certified by peer review) is the author/funder, who has granted medRxiv a license to display the preprint in perpetuity.
It is made available under a CC-BY 4.0 International license .

**Spaniard**	846127	19133 (2.26%)	768999 (90.88%)	11150 (1.32%)	46845 (5.54%)
**All Other Spanish/Hispanic/Latino**	2343763	171293 (7.31%)	1835581 (78.32%)	76926 (3.28%)	259963 (11.09%)

**note:**	
**Total Hispanic**	18.43%
**Total non-Hispanic**	81.57%

[a]N/A refers to individuals who lived in group quarters or housing units that were vacant at the time of data collection
[b]service refers to paying a cell phone company of internet service provider
[c]No responses are regarding to access at home; may not be representative of access to public internet sources
[d]Total Spanish/Hispanic/Latino includes all related origins below (Mexican, Puerto Rican, Cuban, Dominican, Costa Rican, Guatemalan, Honduran, Nicaraguan, Panamanian, Salvadoran, Other Central American, Argentinean, Bolivian, Chilean, Colombian, Ecuadorian, Paraguayan, Peruvian, Uruguayan, Venezuelan, Other South American, Spaniard, All other Spanish/Hispanic/Latino

medRxiv preprint doi: https://doi.org/10.1101/2023.06.22.23291764; this version posted June 29, 2023. The copyright holder for this preprint (which was not certified by peer review) is the author/funder, who has granted medRxiv a license to display the preprint in perpetuity.
It is made available under a CC-BY 4.0 International license .

Table S5b. ACS 1-Year Estimates Access to the Internet in 2021 According to the Public Use Microdata Sample by Hispanic Ethnicity

Source: ACS 1-Year Estimates Public Use Microdata Sample 2021					
Weight used: PWGTP					
**Access to the Internet (ACCESSINET)**					
**Recoded detailed Hispanic origin**	Total	N/A[a]	Yes, by paying a service[b]	Yes, without paying a service	No access[c]
**Total**	331893745	7760859 (2.34%)	301737502 (90.91%)	6328745 (1.91%)	16066639 (4.84%)
**Not Spanish/Hispanic/Latino**	269363212	6755143 (2.51%)	244734198 (90.86%)	5004337 (1.86%)	12869534 (4.78%)
**Total Spanish/Hispanic/Latino[d]**	62530533	1005716 (1.61%)	57003304 (91.16%)	1324408 (2.12%)	3197105 (5.11%)
**Mexican**	37249675	520237 (1.40%)	34070010 (91.46%)	783778 (2.10%)	1875650 (5.04%)
**Puerto Rican**	5801064	105552 (1.82%)	5286988 (91.14%)	131245 (2.26%)	277279 (4.78%)
**Cuban**	2402116	38332 (1.60%)	2166202 (90.18%)	58667 (2.44%)	138915 (5.78%)
**Dominican**	2421393	27246 (1.13%)	2235808 (92.34%)	52818 (2.18%)	105521 (4.36%)
**Costa Rican**	183070	2555 (1.40%)	174907 (95.54%)	2532 (1.38%)	3076 (1.68%)
**Guatemalan**	1770604	18814 (1.06%)	1511965 (85.39%)	40778 (2.30%)	199047 (11.24%)
**Honduran**	1123243	11711 (1.04%)	977720 (87.05%)	27011 (2.40%)	106801 (9.51%)
**Nicaraguan**	452590	4046 (0.89%)	416153 (91.95%)	11921 (2.63%)	20470 (4.52%)
**Panamanian**	235136	4450 (1.89%)	215096 (91.48%)	7403 (3.15%)	8187 (3.48%)
**Salvadoran**	2486513	17928 (0.72%)	2301504 (92.56%)	45154 (1.82%)	121927 (4.90%)
**Other Central American**	27044	665 (2.46%)	25329 (93.66%)	21 (0.08%)	1029 (3.80%)
**Argentinean**	290013	3524 (1.22%)	271612 (93.66%)	7778 (2.68%)	7099 (2.45%)
**Bolivian**	127381	576 (0.45%)	122686 (96.31%)	1565 (1.23%)	2554 (2.01%)
**Chilean**	191055	3352 (1.75%)	179861 (94.14%)	3518 (1.84%)	4324 (2.26%)
**Colombian**	1422553	17776 (1.25%)	1342697 (94.39%)	29947 (2.11%)	32133 (2.26%)
**Ecuadorian**	831552	7443 (0.90%)	782190 (94.06%)	10683 (1.28%)	31236 (3.76%)
**Paraguayan**	29244	745 (2.55%)	27958 (95.60%)	265 (0.91%)	276 (0.94%)
**Peruvian**	707922	7124 (1.01%)	667862 (94.34%)	13247 (1.87%)	19689 (2.78%)
**Uruguayan**	63877	1425 (2.23%)	58747 (91.97%)	1748 (2.74%)	1957 (3.06%)
**Venezuelan**	643470	4542 (0.71%)	610146 (94.82%)	9250 (1.44%)	19532 (3.04%)
**Other South American**	40927	232 (0.57%)	39126 (95.60%)	535 (1.31%)	1034 (2.53%)
**Spaniard**	992848	17120 (1.72%)	931401 (93.81%)	16244 (1.64%)	28083 (2.83%)
**All Other Spanish/Hispanic/Latino**	3037243	190321 (6.27%)	2587336 (85.19%)	68300 (2.25%)	191286 (6.30%)

note:

medRxiv preprint doi: https://doi.org/10.1101/2023.06.22.23291764; this version posted June 29, 2023. The copyright holder for this preprint (which was not certified by peer review) is the author/funder, who has granted medRxiv a license to display the preprint in perpetuity.
It is made available under a CC-BY 4.0 International license .

**% total Hispanic**	18.84%
**% total non-Hispanic**	81.16%

[a]N/A refers to individuals who lived in group quarters or housing units that were vacant at the time of data collection
[b]service refers to paying a cell phone company of internet service provider
[c]No responses are regarding to access at home; may not be representative of access to public internet sources
[d]Total Spanish/Hispanic/Latino includes all related origins below (Mexican, Puerto Rican, Cuban, Dominican, Costa Rican, Guatemalan, Honduran, Nicaraguan, Panamanian, Salvadoran, Other Central American, Argentinean, Bolivian, Chilean, Colombian, Ecuadorian, Paraguayan, Peruvian, Uruguayan, Venezuelan, Other South American, Spaniard, All other Spanish/Hispanic/Latino

medRxiv preprint doi: https://doi.org/10.1101/2023.06.22.23291764; this version posted June 29, 2023. The copyright holder for this preprint (which was not certified by peer review) is the author/funder, who has granted medRxiv a license to display the preprint in perpetuity.
It is made available under a CC-BY 4.0 International license .

Figure S1. Clinical Sites of The DCM Consortium for the DCM Precision Medicine Study.

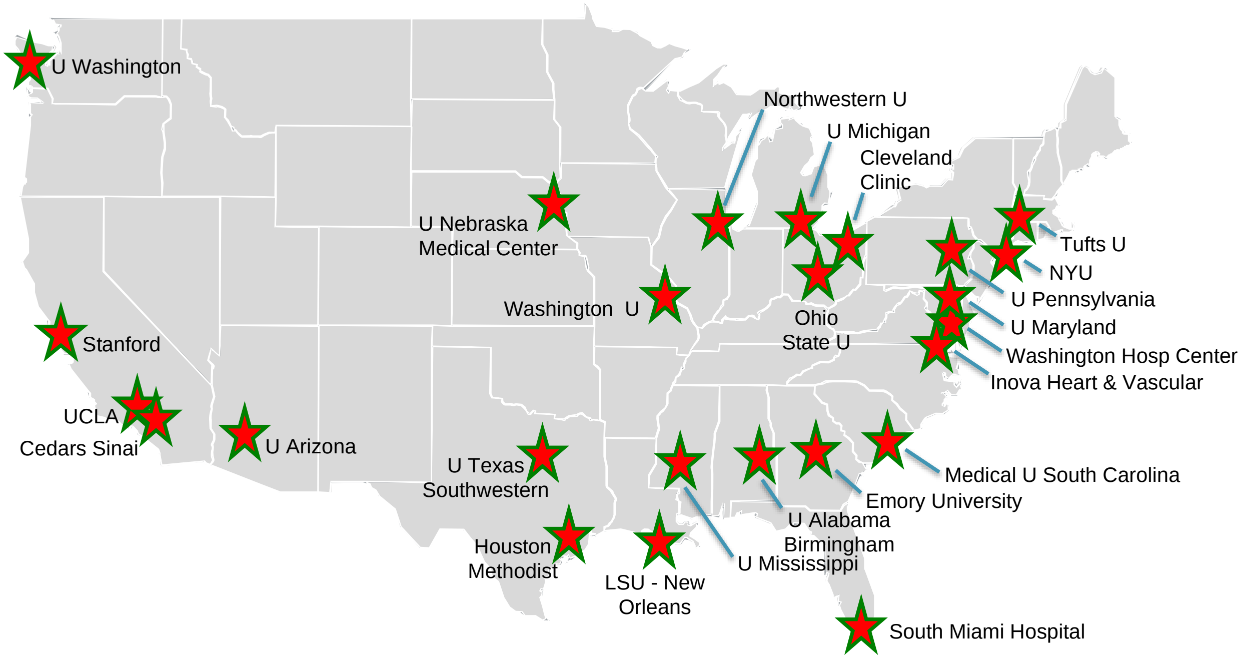

Shown are the 25 clinical sites of the DCM Consortium who enrolled probands and family members for the DCM Precision Medicine Study. The Ohio State University site served as an enrolling site as well as the coordinating center.

medRxiv preprint doi: https://doi.org/10.1101/2023.06.22.23291764; this version posted June 29, 2023. The copyright holder for this preprint (which was not certified by peer review) is the author/funder, who has granted medRxiv a license to display the preprint in perpetuity.
It is made available under a CC-BY 4.0 International license .

Figure S2. Beta testing phases of The DCM Project Portal

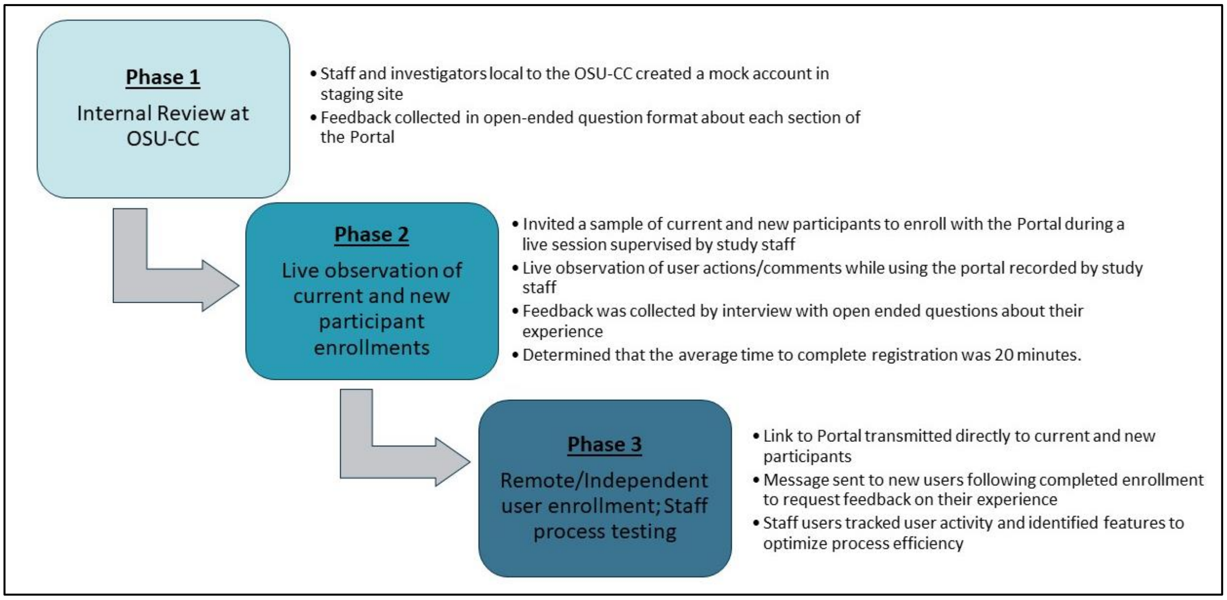

Figure S2. Three phases of beta testing were conducted before opening the DCM Project Portal for active service. Phase 1 included an internal review of staff and investigators familiar with the research project. Staff and investigators created mock accounts and offered feedback in an open-ended question form. After incorporating feedback, Phase 2 included a sample of participant users, including those previously consented to the study as well as newly invited. Individuals were observed going through the portal process live while a staff took notes of the actions and comments made during the experience. Feedback was collected directly once the process was completed by structured interview by the observing study staff member. Adjustments were made according to Phase 2 results before beginning Phase 3, where select participants (new and previously consented) were invited to create an account but this time independently. Progress was tracked by staff and feedback collected through the messaging system upon completion. Phase 3 findings included requests from both users and staff to maximize usability and efficiency.
